# Effect of Friction Stir Welding Parameters on Mechanical Properties and Formability of Pre-Hardened 2219 Aluminum Alloy

**DOI:** 10.3390/ma19091855

**Published:** 2026-04-30

**Authors:** Xiaoming Ye, Xianlong Meng, Qiu Pang, Sujia Zhang

**Affiliations:** 1 State Key Laboratory of Light Superalloys, Wuhan University of Technology, Wuhan 430070, China; yexm@dfmc.com.cn (X.Y.); mengxl@whut.edu.cn (X.M.); 2 Research Institute of Dongfeng Motor Group Co., Ltd., Wuhan 430058, China; 3 Department of Mechanical and Electrical Engineering, Wuhan Donghu University, Wuhan 430212, China; 4 Shanghai Academy of Spaceflight Technology, Shanghai 201109, China

**Keywords:** friction stir welding, Al–Cu–Mn alloy, welding parameters, mechanical properties, formability

## Abstract

**Highlights:**

- Forward speed is the dominant parameter affecting mechanical properties of PH welded sheets.
- The excellent strength–ductility synergy in the PH welded sheets was attributed to a unique microstructural mechanism: the cooperative precipitation of θ″ and θ′ phases effectively hindered dislocation motion, while a high geometric compatibility factor (m′ > 0.7) promoted the activation of multiple slip systems and dislocation rearrangement.
- This study elucidates the microstructural mechanisms underlying the synergistic improvement in both strength and ductility of PH welded sheets under a process that combines FSW and PHF techniques.
- Offers critical guidelines for optimizing FSW parameters (specifically forward speed) to maximize the mechanical performance of PH alloys.

**Abstract:**

In this study, the effects of friction stir welding (FSW) parameters on the mechanical properties and formability of pre-hardened (PH) 2219 aluminum alloy welds were systematically investigated through tensile testing and Erichsen tests. Energy dispersive spectrometry (EDS), electron back scatter diffraction (EBSD), and a transmission electron microscope (TEM) were employed to characterize the microstructure of the PH alloy weld joints, revealing the strength–ductility synergy mechanism of the PH welded sheets. Experimental results indicated that with respect to mechanical properties, when the welding rotational speed was fixed at 1000 rpm, increasing the forward speed from 50 mm/min to 150 mm/min reduced the ultimate tensile strength (UTS) by 6.3% and decreased the EL by 21.4%. When the forward speed was fixed at 50 mm/min, increasing the rotational speed from 500 rpm to 1500 rpm resulted in only a 0.4% variation in UTS and maintained a stable EL, demonstrating that forward speed is the dominant parameter affecting mechanical properties. In terms of formability, at a lower forward speed (50 mm/min), the Erichsen value exhibited a single-peak trend with increasing rotational speeds. At higher forward speeds (100 or 150 mm/min), the Erichsen value was insensitive to changes in rotational speed. When the rotational speed was fixed at 1500 rpm, increasing the forward speed from 50 mm/min to 150 mm/min reduced the Erichsen value by 21.3%. Microstructural strengthening mechanism: In the weld zone, the cooperative precipitation of the θ″ and θ′ phases effectively hindered dislocation motion. Simultaneously, the high geometric compatibility factor promoted the activation of multiple slip systems, and dislocation rearrangement subsequently led to the formation of sub-grain boundaries, thereby achieving strength–ductility cooperation. These findings provide theoretical support for the performance-driven welding process design of high-strength aluminum alloy components in aerospace applications.

## 1. Introduction

High-strength aluminum alloy welded sheets, with their excellent lightweight and high-strength properties, are promising for applications in the automotive and aerospace industries [1,2,3,4]. However, when employing traditional fusion welding processes for high-strength aluminum alloy welded sheets, it is often difficult for the joints to achieve the required performance levels [5]. This is primarily due to the susceptibility to metallurgical defects such as porosity and solidification cracking during the welding process. Furthermore, affected by the welding thermal cycle, the strength of the joints decreases significantly compared to the base material [6,7]. Friction stir welding (FSW) is an advanced solid-temper joining technology that effectively prevents metallurgical defects and preserves material properties and has therefore been widely used to join aerospace alloys such as 2219 aluminum [8,9,10].

The FSW process parameters determine the heat input during welding, which exerts a significant influence on the strength and ductility of the welded sheet joints. Kalinenko et al. [11] found that superior welding results could be achieved when the rotational speed was controlled at ≥1000 rpm and the forward speed at ≤150 mm/min. In contrast, Zhang et al. [12] observed that even with the forward speed controlled at 100 mm/min, a rotational speed of 1650 rpm still led to the formation of a wider thermo-mechanically affected zone (TMAZ) due to increased heat input, resulting in a decline in the performance of the joint. Therefore, the selection of welding parameters typically relies on extensive trial-and-error or empirical accumulation. Kumar et al. [13] developed a three-dimensional computational fluid dynamics (CFD) model to analyze heat input and material flow during the welding process. They determined that the optimal process parameters ranged from a rotational speed of 1000–1600 rpm to a forward speed of 63 mm/min, significantly reducing the time cost associated with parameter selection for the welded sheet joints.

However, the temperature gradients inherent in the FSW process cause material softening, leading to a decline in the mechanical properties of the joints. To restore the performance of the joints, post-weld heat treatment is typically required. Alexander Kalinenko et al. [14] pointed out that post-weld heat treatment can effectively recover the strength loss in the joints caused by abnormal grain growth (AGG). Sharma V et al. [15] observed that friction stir welded joints of AA5083 and AA2014-T651 aluminum alloys exhibited excellent mechanical properties after artificial aging at 175 °C. The effectiveness of heat treatment depends heavily on the selection of process parameters. Baghdadi et al. [16] confirmed that solution treatment of Al–Mg–Si alloy FSW joints at 535 °C for 1 h, followed by artificial aging at 175 °C for 8 h, could restore their strength and elongation to the levels of the base material (BM) in the T6 temper. Aydın et al. [17] reported that the hardness of the thermo-mechanically affected zone (TMAZ), heat affected zone (HAZ), and BM of the joints can be significantly improved through artificial aging at 190 °C for 10 h.

Although traditional solution treatment and aging have been shown to effectively improve the mechanical properties of friction stir welded joints, they have significant drawbacks. Singh et al. [18] subjected AA2014-T651/7075-T651 welds to 24 h of low-temperature treatment and found that post-weld low-temperature heat treatment had a limited effect on strength. Pabandi et al. [19] increased the ultimate tensile strength by 24.4% through post-weld heat treatment, but the heat treatment lasted as long as 19 h. Hu et al. [20] point out that although post-weld heat treatment can improve weld strength, this comes at the expense of ductility; research by Yang et al. [21] also confirms this. As can be seen, traditional heat treatment processes not only involve long production cycles but also face the challenge of balancing strength and ductility.

To overcome the aforementioned challenges, this study adopted the pre-hardened forming (PHF) technique proposed by Hua et al. [22] for high-strength aluminum alloys. This process first subjects the welded sheets to a short-duration post-weld heat treatment (including high-temperature solution treatment, quenching, and aging) to bring them to a PH temper. At this stage, the ductility of the sheets is comparable to that of an annealed temper, while the ultimate tensile strength (UTS) approaches the peak age-hardening strength, thereby achieving a synergistic improvement in both strength and ductility and significantly reducing heat treatment time. Subsequently, the PH sheets are directly formed using dies, achieving both shape formation and performance enhancement simultaneously; the final components reach peak aging strength without requiring any subsequent heat treatment. The PHF process offers significant advantages: it eliminates the need for subsequent heat treatment, ensures high dimensional accuracy through die forming (far superior to conventional processes), and achieves an extremely short manufacturing cycle.

Uniaxial tensile and Erichsen tests were performed on PH welded sheets produced with various welding parameters to determine how these parameters affect the mechanical properties and formability of 2219 aluminum alloy welded sheets. The specimen exhibiting the best overall performance was selected for microstructural analysis to identify the mechanisms responsible for its improved properties. This work aims to optimize processing conditions and achieve both enhanced performance and a shortened manufacturing cycle.

## 2. Experiments

### 2.1. Experimental Materials

In this study, the material used was 2219 aluminum alloy with a sheet thickness of 1.5 mm. The initial temper of the sheets was rolled, and their chemical composition is shown in Table 1 [23]. The original rolled sheets were cut into small pieces of 250 mm × 60 mm × 1.5 mm for the subsequent welding process.

**Figure 1:**
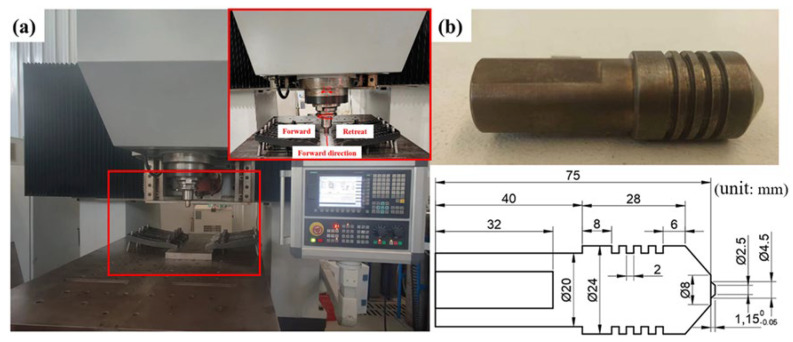
FSW test tools: (**a**) NFSW-650 FSW equipment; (**b**) stir tool dimensions.

As shown in Figure 1a, the FSW experiment was conducted on an NFSW-650 machine. The stir tool used in the experiment and its dimensions are shown in Figure 1b. The two identical 2219 aluminum alloy sheets were fixed on a backing sheet. During welding, the rotating tool generated heat and pressure, causing the material to plasticize and form the weld seam as it cooled. Since FSW is a solid-temper joining method that does not involve the melting of the base material, or require filler metal or shielding gas, it effectively avoids issues such as large deformations, cracks, and porosity. The welding quality is primarily influenced by the welding rotational speed and forward speed. In the experiments, these were used as variables, and the parameters were set according to Table 2 to study their impact on the performance of PH welded joints of 2219 aluminum alloy. In addition to the welding forward speed and rotational speed, other constant FSW parameters were maintained as follows: a tool tilt angle of 2.5°, a plunge depth of 0.2 mm, and a dwell time of 10 s. The welding process was executed under position control mode with zero tool offset. To ensure stability, the specimens were secured using professional fixtures on an H13 steel backing sheet.

The PHF process for 2219 aluminum alloy FSW welded sheets involves solution treatment and artificial aging to produce welded sheets in an underaged condition. Compared to the solution-treated W-temper and the fully aged T6-temper, this temper not only improves strength but also maintains good plasticity. The solution treatment was carried out according to the standard YS/T 591-2017, with all PH welded sheets having the same solution treatment parameters: holding at 540 °C for 40 min, followed by rapid water quenching. The artificial aging time is a key factor in enhancing strength during the PHF process. Based on the results of preliminary exploratory tests, the aging parameters of 150 °C for 12 h were ultimately selected [23]. The 2219 aluminum alloy PH welded sheets were subjected to artificial aging in an electric blast drying oven, with a temperature control accuracy of ±1 °C, a temperature range of +5 °C to 300 °C, and a temperature uniformity of ±2%.

### 2.2. Performance Testing Methods for PH Welded Sheets

**Figure 2:**
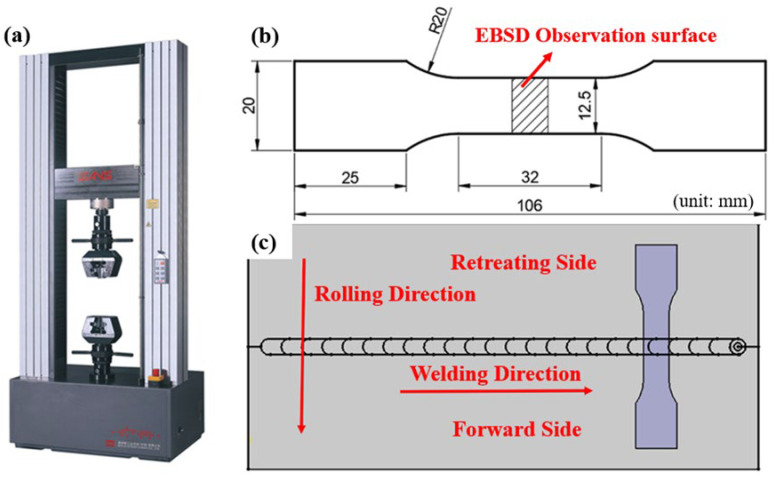
Tensile testing machine and tensile specimen: (**a**) SANS CMT 5205 electronic universal testing machine; (**b**) tensile specimen dimensions; (**c**) location of tensile specimen selection.

The uniaxial tensile test on the 2219 aluminum alloy PH welded sheets was conducted using the SANS CMT 5205 electronic universal testing machine to evaluate their mechanical properties, as shown in Figure 2a. The stress and displacement data were processed using the MTS Test-Suite software (Power Test V3.0C) to plot the engineering stress–strain curve. The dimensions of the tensile specimen were designed according to the standard GB/T 228.1-2021, as shown in Figure 2b. Tensile specimens were obtained from the PH welded sheets by wire electrical discharge machining, ensuring that the tensile direction was perpendicular to the weld seam and parallel to the rolling direction, as shown in Figure 2c. The tensile speed was set to 1.5 mm/min. To ensure the accuracy of the results, the average value of three specimens tested under each welding parameter condition was used as the final mechanical performance indicator.

**Figure 3:**
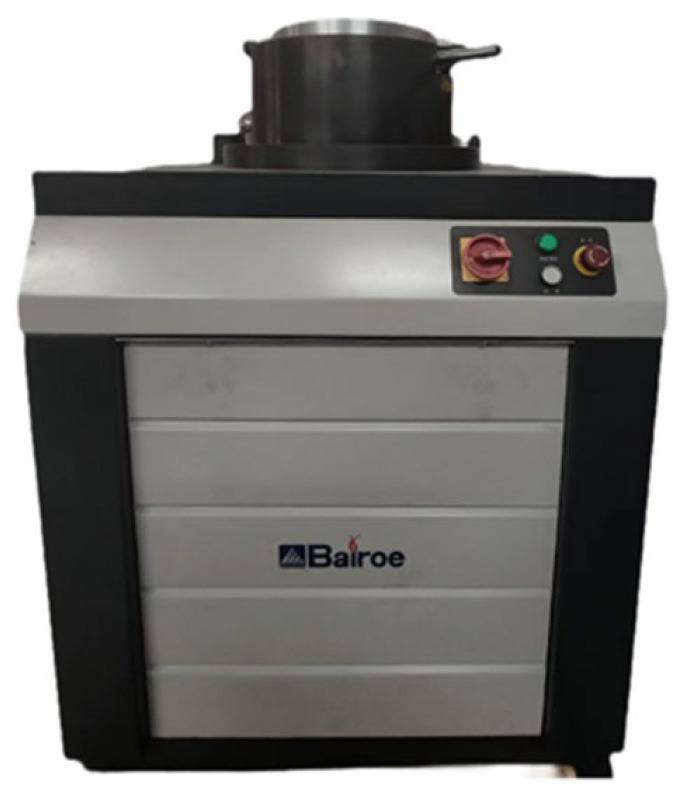
Computer-controlled sheet metal forming testing machine.

The Erichsen test is a standard method for evaluating the formability of metal sheets. It quantitatively assesses formability by measuring the material′s ability to plastically deform without cracking. A higher Erichsen value indicates better formability. According to the standard GB/T 4156-2020, the Erichsen test specimen diameter for PH welded sheets was set to 90 mm, with the weld seam located at the center of the specimen without any offset. The tests were conducted using a BTP-300 microcomputer-controlled sheet metal forming testing machine (Hongpu, Shanghai, China), as shown in Figure 3.

### 2.3. Microstructure Analysis

The microstructure includes the crystal structure, phase interfaces, and precipitate distribution, which determine the mechanical properties of the material. In this study, the microstructure of the central region (weld zone) of the tensile specimen of PH welded sheets was analyzed, and the effect of microstructure on the mechanical properties of PH welded sheets was analyzed using electron back scatter diffraction (EBSD) and energy dispersive spectrometry (EDS) under the JSM-IT800 thermal field emission scanning electron microscope (JEOL Ltd., Tokyo, Japan). The obtained specimens were polished and ground using an MP-2B metallographic specimen polishing machine (Hongpu, Shanghai, China). During the grinding process, the grit size of the sandpaper ranged from 800 to 3000 (CW). The polishing treatment of the specimens involved initial polishing with aluminum oxide polishing agent, followed by water polishing to achieve a mirror finish. Before EBSD characterization, the specimen surface was electropolished to improve quality. For EDS analysis, the main detection elements for 2219 aluminum alloy are Al, Cu, Mn, Mg, Si, and Fe. X-ray spectra were collected at a magnification of 1000× at the protruding positions during point scans.

## 3. Results

### 3.1. Effect of Welding Parameters on the Mechanical Properties of PH Welded Joints

#### 3.1.1. Effect of Welding Rotational Speed on the Mechanical Properties of PH Welded Joints

**Figure 4:**
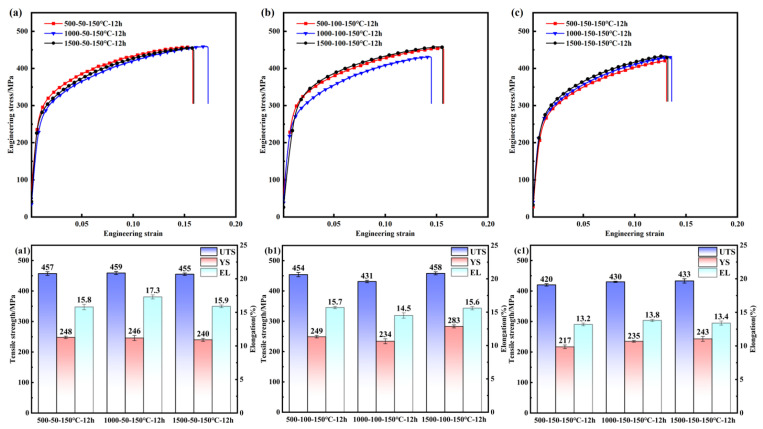
Effect of rotational speed on the mechanical properties of PH welded joints at different forward speeds (v): 50 mm/min, 100 mm/min, 150 mm/min; (**a1**,**b1**,**c1**) corresponding UTS, YS, and EL values for the conditions shown in (**a**), (**b**), and (**c**), respectively.

The relationship between the mechanical properties of PH welded joints and the rotational speed when the welding forward speed was held constant is shown in Figure 4. At a forward speed of 50 mm/min, the UTS fluctuated at about 457 ± 2 MPa and the yield strength (YS) at about 244 ± 4 MPa as the rotational speed increased, indicating stable performance. When the forward speed was increased to 100 mm/min, the UTS first decreased and then increased with rotational speed, reaching a peak of 458 MPa at 1500 rpm with a difference in value of 27 MPa; the YS variation became more pronounced with a difference in value of 49 MPa, resulting in unstable performance. At a forward speed of 150 mm/min, the UTS decreased overall to 420–433 MPa (a reduction of 5.6–7.6% compared to 50 mm/min) and the YS dropped to 217–243 MPa (a reduction of 2.1–9.5%), with increasing rotational speed unable to reverse the deterioration. These results indicate that at a low forward speed (50 mm/min), the rotational speed has minimal impact on the mechanical properties of PH welded joints; at a moderate forward speed (100 mm/min), the properties fluctuate with rotational speed; and at a high forward speed (150 mm/min), the mechanical properties deteriorate regardless of the rotational speed.

#### 3.1.2. The Effect of Welding Forward Speed on the Mechanical Properties of PH Welded Joints

**Figure 5:**
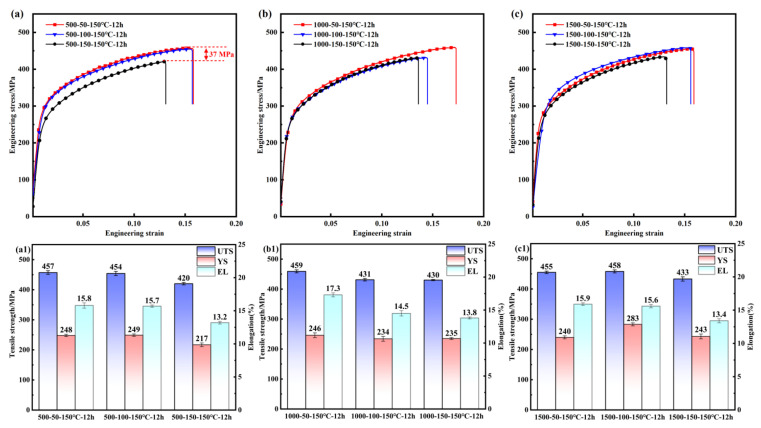
Effect of forward speed on the mechanical properties of PH welded joints at different rotational speeds (w): (**a**) 500 rpm, (**b**) 1000 rpm, (**c**) 1500 rpm; (**a1**,**b1**,**c1**) corresponding UTS, YS, and EL values for the conditions shown in (**a**), (**b**), and (**c**), respectively.

The relationship between the mechanical properties of PH welded joints and the forward speed when the welding rotational speed was held constant is shown in Figure 5. At a rotational speed of 500 rpm, as the forward speed increased from 50 mm/min to 100 mm/min, the UTS remained stable at 455 ± 3 MPa and the YS at 248 ± 1 MPa; however, at a forward speed of 150 mm/min, the UTS decreased to 420 MPa (8.1% lower), and the YS dropped to 217 MPa (12.8% lower). At a rotational speed of 1500 rpm, the UTS was consistent with that at 500 rpm; however, the YS varied significantly, reaching a peak of 283 MPa at a forward speed of 100 mm/min and a minimum of 240 MPa at 50 mm/min, with a difference in value of 43 MPa. At a rotational speed of 1000 rpm, as the forward speed increased, the UTS decreased from 459 MPa to 430 MPa (a reduction of 6.3%), while the YS decreased from 246 MPa to 235 MPa (a reduction of 4.4%). These results indicate that within the rotational speed range of 500–1500 rpm, maintaining a lower forward speed (50 mm/min) results in stable and excellent mechanical properties of PH welded joints, whereas increasing the forward speed to 150 mm/min leads to a significant deterioration in mechanical performance.

### 3.2. Effect of Welding Parameters on the Formability of PF Welded Sheets

#### 3.2.1. The Effect of Welding Rotational Speed on the Formability of PH Welded Sheets

**Figure 6:**
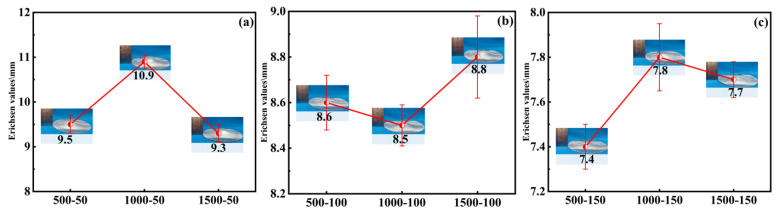
The effect of rotational speed on Erichsen values at different forward speeds (v): (**a**) 50 mm/min, (**b**) 100 mm/min, (**c**) 150 mm/min.

With the welding forward speed held constant, the Erichsen values of PH welded sheets at different rotational speeds are shown in Figure 6. The effect of rotational speed on the formability of PH welded sheets can be divided into two cases: (1) At a low forward speed (50 mm/min), the formability first increased and then decreased with increasing rotational speed, reaching a peak value of 10.9 mm at 1000 rpm and falling to a minimum value of 9.3 mm at 1500 rpm, a reduction of 14.6%. (2) At higher forward speeds (100 mm/min and 150 mm/min), the formability did not change significantly with increasing rotational speed; however, compared to the formability at 50 mm/min, it was clearly reduced, with a decrease ranging from 5.3% to 32.1%.

For the first case, at a lower forward speed (e.g., 50 mm/min), the extended dwell time of the stir tool in the weld seam area increased the total heat input. During this process, as the rotational speed increased, an intensification of plastic deformation and frictional heating was observed, which promoted dynamic recrystallization. Consequently, a fine and equiaxed grain structure was formed, and the enhanced ductility significantly improved the formability of the weld seam region. In the PH process, the solution-aging treatment is designed to dissolve the alloying elements and then precipitate them in a controlled manner to enhance strength and, to some extent, ductility [22]. Initially, the increased heat input from a higher rotational speed provided conditions favorable for a more uniform solution treatment during post-weld heat treatment, leading to an optimal distribution of precipitates during aging. However, exceeding a certain rotational speed resulted in overheating, which induced an overaging effect during post-weld heat treatment; this may lead to precipitate coarsening and grain growth, thereby reducing both strength and formability.

For the second case, when the forward speed was higher (e.g., 100 mm/min and 150 mm/min), the stir tool moved faster across the material. This reduced the time for heat accumulation in any given area and resulted in a milder thermal cycle that limited the weld zone from reaching peak temperatures. At these higher forward speeds, the faster movement produced a more balanced microstructure, making it less sensitive to changes in rotational speed. The PH treatment (solution-aging) is designed to homogenize the weld zone microstructure and optimize mechanical properties through precipitation strengthening. When the initial differences in microstructure and residual stress caused by different rotational speeds are small (as at higher forward speeds), the heat treatment can more uniformly restore or enhance the weld seam′s formability, thereby reducing its dependence on the initial welding conditions. Consequently, the milder and more uniform heat input at higher forward speeds led to less variation in microstructural evolution during welding, resulting in more consistent formability after PH processing across different rotational speeds.

#### 3.2.2. Effect of Welding Forward Speed on the Formability of PH Welded Sheets

**Figure 7:**
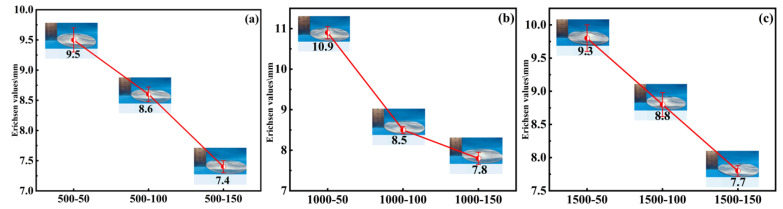
The effect of forward speed on Erichsen values at different rotational speeds (w): (**a**) 500 rpm, (**b**) 1000 rpm, (**c**) 1500 rpm.

With a constant welding rotational speed, the variation of Erichsen values with forward speed for PH welded sheets is shown in Figure 7. When the rotational speed was fixed, the formability of PH welded sheets exhibited a negative correlation with forward speed. Specifically, at a rotational speed of 1000 rpm, the Erichsen value decreased from 10.9 mm to 7.8 mm as the forward speed increased. At this time, the maximum decrease reached 28.4%, whereas at 1500 rpm, the Erichsen value decreased from 9.8 mm to 7.7 mm, and this time the smallest decrease reached 21.4%. This behavior is attributed to the fact that welding forward speed directly influences the heat input and cooling rate in the weld seam area. Higher forward speeds shorten the dwell time of the stir tool in the weld seam area, resulting in faster cooling and lower heat input. Since the precipitation hardening mechanism of 2219 aluminum alloy is sensitive to thermal cycles, these cycles significantly affect the microstructure after post-weld heat treatment and, consequently, the mechanical properties. At higher forward speeds, the reduced heat input may be insufficient to achieve a uniform solution temper prior to aging; incomplete dissolution of precipitates or a non-uniform microstructure may lead to an uneven aging response, thereby diminishing the overall formability of the PH welded sheets.

### 3.3. Effect of Welding Heat Input on the Mechanical Properties of PH Welded Joints

It can be seen from the above experimental results that the mechanical properties of PH welded joints did not exhibit a simple linear variation with either rotational speed or forward speed. Specifically, the influence of rotational speed on the mechanical performance of the joints showed distinct directional discrepancies under different forward speed conditions. Similarly, the impact of forward speed on the properties of the joints was mutually constrained by the rotational speed. Therefore, when analyzing the effect of welding parameters on the mechanical properties of the PH welded joints, the synergistic effect of rotational and forward speeds must be comprehensively considered. In the FSW process, the peak welding temperature is closely correlated with the heat input, and their relationship can be expressed by Equation (1) [24]:

(1) $$\frac{T}{T_{m}}\;=\;K{\left(\frac{w^{2}}{v\;\times {\;10}^{4}}\right)}^{\alpha }$$

Herein, T (°C) represents the peak temperature during welding. T_m_ (°C) denotes the melting point of the welded alloy, w is the rotational speed (rpm), and v is the welding forward speed (mm/min). K and α are constants. K typically ranges from 0.65 to 0.75, and α ranges from 0.04 to 0.06 [25]. According to Equation (1), when the welding environment remains constant, T_m_, K, and α are all fixed values. Under such circumstances, the peak welding temperature depends solely on the ratio w^2^/v. Therefore, in this physical model, w^2^/v is defined as the heat index (HI) for welding. The HI values corresponding to different welding parameters are presented in Table 3.

It should be noted that while the HI based on the empirical model provides a theoretical framework for estimating peak welding temperatures, no direct thermal measurements (e.g., via thermocouples or pyrometers) were performed in this study to explicitly validate these values. This represents a limitation of the current experimental setup. Nevertheless, the HI proxy serves as a widely accepted qualitative indicator for evaluating the relative heat input per unit length under various welding parameters, as supported by the study by Fu et al. [26]. Future research incorporating temperature monitoring will be essential to further refine and calibrate the constants K and α for specific PH-treated alloy systems.

**Figure 8:**
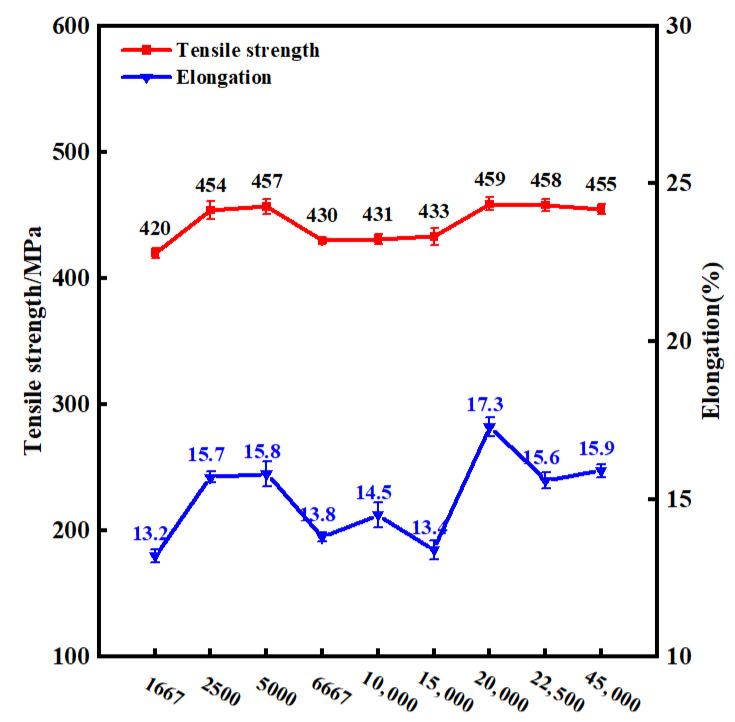
Tensile strength and elongation curves of PH welded joints as a function of HI.

Taking the HI as the independent variable and the tensile strength and elongation as the dependent variables, the evolution of mechanical properties of PH welded joints with HI was obtained and plotted in Figure 8. The results demonstrate that the relationships between tensile strength, elongation, and the HI are not simply linear. The experimental data clearly indicate that both the tensile strength and elongation of the joints declined at the extremely low and extremely high ends of the HI range. The joint achieved the optimal comprehensive mechanical properties at HI = 20,000 (i.e., w = 1000 rpm, v = 50 mm/min). By contrast, the overall performance of the PH welded joints was significantly degraded under low or medium HI conditions. It is noteworthy that the minima of both tensile strength and elongation occurred in the three parameter groups with HI = 1667, 6667, and 15,000. Analysis reveals that these three groups shared a common feature: a forward speed of 150 mm/min. This observation strongly proves that, for PH welded joints, the effect of forward speed on the joints’ mechanical properties is considerably more significant than that of the rotational speed.

With the initial increase in HI, as it reached an appropriate level, the welding thermal cycle effectively promoted plastic deformation and dynamic recrystallization, thereby forming a fine and uniform equiaxed grain structure in the weld zone. Under loading conditions, this microstructure facilitated a more uniform distribution of stress. Furthermore, a suitable HI provided a more homogeneous microstructure for subsequent solution treatment, promoting the uniform precipitation of precipitates during the aging process and thus significantly enhancing the comprehensive mechanical properties of the material. However, when the HI exceeded a certain critical value, an excessively high HI induced overaging, leading to the coarsening of precipitates, thereby weakening its strengthening effect and reducing the strength and plasticity of the material. It is worth noting that within a relatively high HI range, a specific thermal cycle window may still exist. Within this window, mechanisms such as the initiation of secondary dynamic recrystallization, modification of precipitation behavior, or elimination of dislocation structures introduced under low HI conditions could theoretically generate a microstructure that simultaneously enhances both strength and ductility. However, if the HI continues to increase beyond this window, its detrimental effects on the microstructure will become dominant, ultimately leading to a decline in the comprehensive mechanical properties of the material.

### 3.4. Mechanical Properties of 2219 Aluminum Alloy W-Temper and PH Welded Sheets

**Figure 9:**
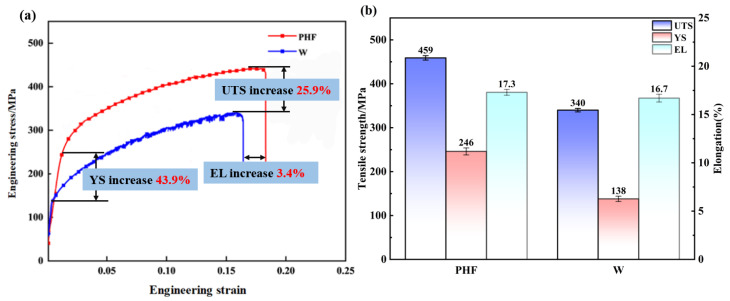
Mechanical properties of PH welded sheets and W-temper welded sheets: (**a**) tensile curve; (**b**) performance comparison chart.

Based on the optimization of welding parameters discussed in Section 3.1, Section 3.2 and Section 3.3, the optimal parameter set (1000 rpm and 50 mm/min) was selected for further investigation. These FSW parameters align with the findings of mainstream research [27,28]. The mechanical properties of the joints in the PH welded sheets and W-temper welded sheets were compared via tensile testing, as illustrated in Figure 9. Notably, to systematically unveil the underlying physical mechanisms governing the performance enhancement, the subsequent microstructural evolution analyses (including EBSD and transmission electron microscopy) were also conducted on the specimens produced under this optimized condition. The test results showed that the UTS of the PH welded sheet was 459 MPa, which was 119 MPa (25.9%) higher than that of the W-temper welded sheet of 340 MPa. The YS reached 246 MPa, an increase of 108 MPa (43.9%) compared to 138 MPa for the W-temper welded sheet. In addition, the EL increased to 17.3%, an increase of 0.6% (3.4%) compared to the 16.7% observed with the W-temper welded sheet. These findings suggest that the PHF process significantly improves the strength and ductility of the welded sheet.

### 3.5. The Effect of Welding Parameters on Fracture Surface Morphology

**Figure 10:**
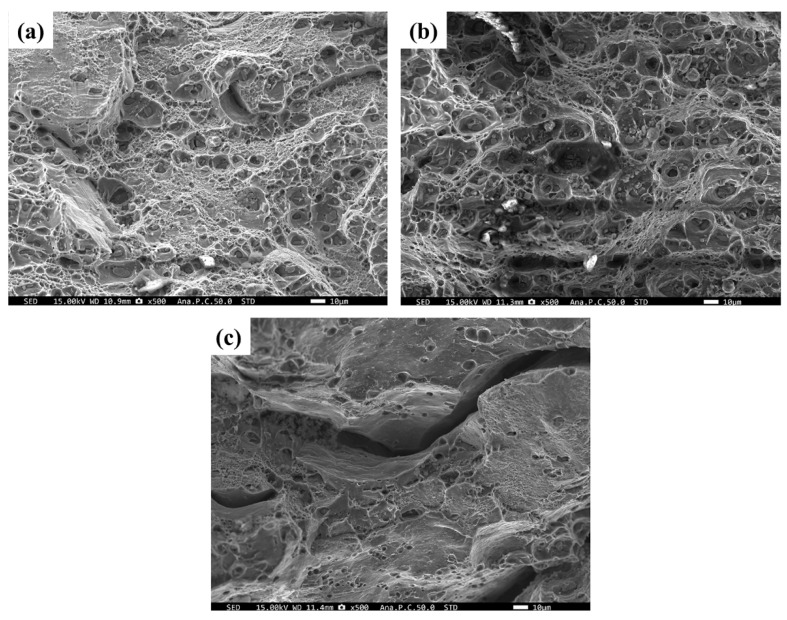
Fracture morphologies of the PH and W-temper welded sheets: (**a**) PH joint (1000 rpm-50 mm/min); (**b**) W-temper joint; (**c**) PH joint (1000 rpm-150 mm/min).

Figure 10 shows that under the welding parameters of 1000 rpm-50 mm/min, the fracture features were similar to those of the W-temper. A large number of uniformly distributed and deep dimples were observed on the fracture surface. This indicates that under this heat input, the solidification-induced particles were sufficiently fragmented. Furthermore, the precipitates effectively stabilized the substructure, and the fracture mode exhibited typical ductile fracture. Under the welding parameters of 1000 rpm-150 mm/min, distinct cracks were observed on the fracture surface, accompanied by a significant reduction in the number of dimples. This indicates that with the increase in welding forward speed, the changes in heat input and plastic flow induced stress concentration or micro-defects within the microstructure, leading to a transition in the fracture mode toward brittle fracture.

## 4. Discussion

### 4.1. Strengthening of Mechanisms

**Figure 11:**
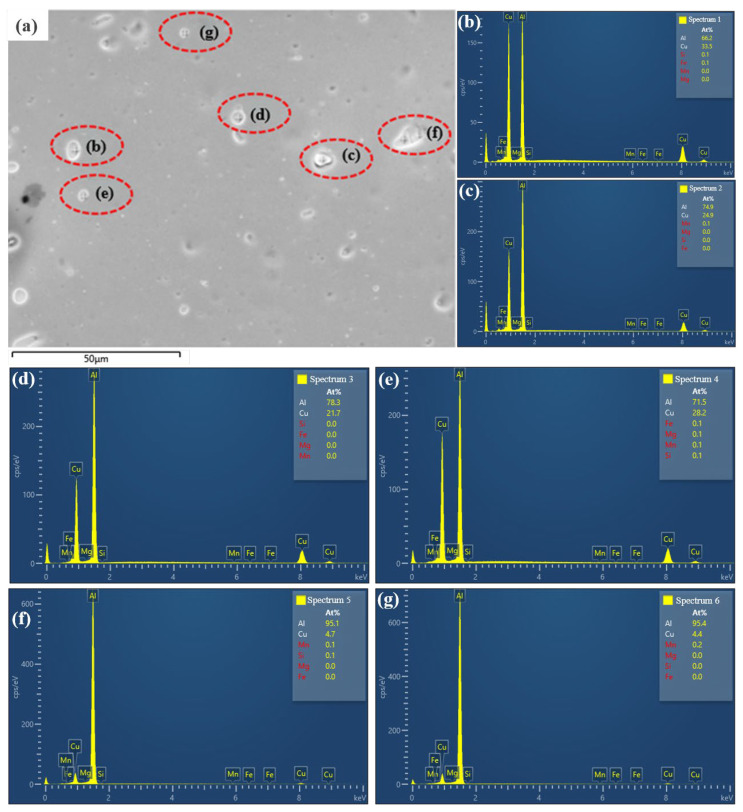
SEM image and corresponding EDS spectra of the weld seam in the PH welded joint: (**a**) SEM image of the weld seam; (**b**) EDS spectrum of particle b; (**c**) EDS spectrum of particle c; (**d**) EDS spectrum of particle d; (**e**) EDS spectrum of particle e; (**f**) EDS spectrum of particle f; (**g**) EDS spectrum of particle g.

**Figure 12:**
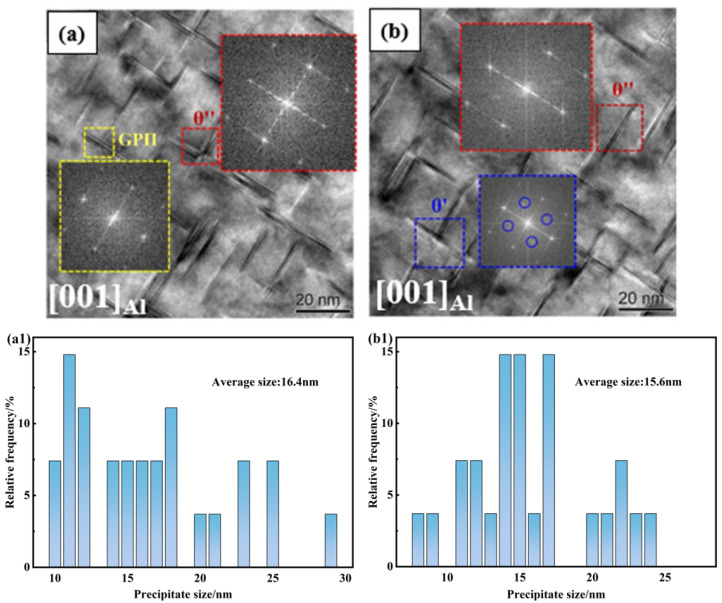
(**a**,**b**) TEM image of the weld seam in a fractured specimen of the PH welded joint; (**a1**,**b1**) particle size distribution graphs corresponding to (**a**) and (**b**), respectively.

During the heat treatment of 2219 aluminum alloy, the precipitation sequence was as follows: GP zones → θ″ → θ′ → θ. Among these, the nanometer-scale θ″ and θ′ phases served as the primary strengthening phases [20,29]. Figure 11 presents the SEM and EDS analysis results at the weld center of the PH welded sheet. It can be observed that coarse micron-sized secondary phase particles are distributed within this region. Elemental analysis reveals an Al: Cu atomic ratio close to 2:1. Combined with the size characteristics, these are identified as primary θ phases that were not fully dissolved during the welding or solution treatment processes. These coarse particles are not precipitation products from aging. Due to their large size and incoherency with the matrix, they tend to induce stress concentration during tensile deformation, serving as crack initiation sites. In contrast, the TEM images in Figure 12 reveal typical nanometer-scale aging precipitation characteristics. Selected area electron diffraction (SAED) patterns confirm the coexistence of GP II zones and θ″, and θ′ phases within the matrix. The fine and coherent GP II zones significantly enhanced the yield strength through the “dislocation cutting” mechanism. In contrast, the θ″ and θ′ phases primarily provided strengthening via the “dislocation bypassing (Orowan)” mechanism. The dispersive distribution of these nanometer-scale strengthening precipitates is the fundamental reason for the high strength achieved in the joints.

### 4.2. Deformation Coordination

**Figure 13:**
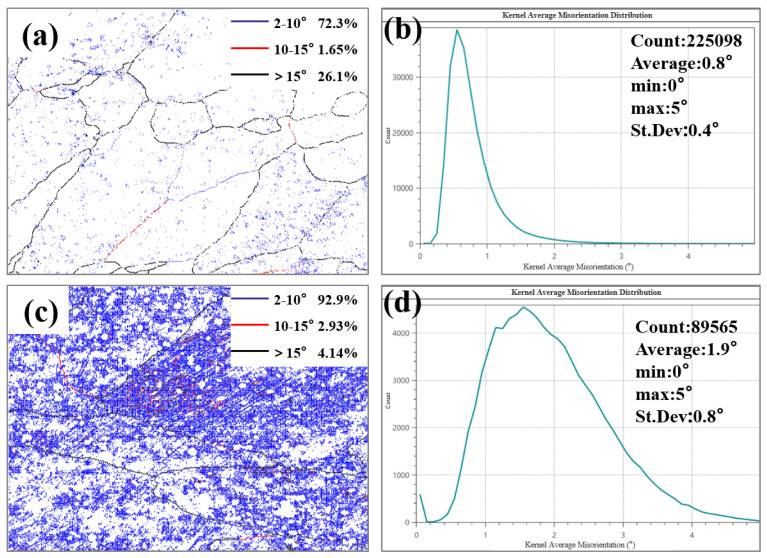
Grain boundary maps and KAM distribution maps of PH and W-temper welded joints: (**a**,**b**) PH welded joint; (**c**,**d**) W-temper welded joint.

The dislocation density and the proportion of high-angle grain boundaries in the weld microstructure are critical factors influencing its ductility. Figure 13 presents the kernel average misorientation (KAM) maps and grain boundary character distributions of the weld regions in both W-temper and PH welded sheets. The average local misorientation angle reflects the dislocation density: a larger average local misorientation angle indicates a higher dislocation density, leading to fewer dislocations being consumed during deformation, thereby reducing ductility. High-angle grain boundaries are beneficial in impeding crack propagation; a higher proportion of high-angle grain boundaries correlates with improved ductility.

The average local misorientation angle in the weld seam of the PH welded joint was 0.8°, whereas in the W-temper welded joint, it was 1.9°, significantly higher than that of the PH weld seam. The proportion of high-angle grain boundaries in the PH welded joint was 26.1%, while in the W-temper joint, it was only 4.14%. Based on these analyses, the ductility of the PH welded joint should be superior to that of the W-temper joint. However, the ductility of the W-temper joint was comparable to that of the PH joint. The primary reason is that the Portevin-Le Chatelier (PLC) effect effectively mitigates localized stress concentration [30]. Upon solution treatment and quenching, the 2219 aluminum alloy forms a supersaturated solid solution. During subsequent plastic deformation, the interaction between the supersaturated solid solution and mobile dislocations triggers dynamic strain aging (DSA), which manifests as serrated flow on the tensile curves, known as the PLC effect. Based on the serration characteristics, the PLC effect is generally classified into three types: A, B, and C, among which the type A PLC effect contributes to postponing the onset of material fracture [31]. As illustrated in Figure 9a, the tensile curves corresponding to the W-temper welded sheets exhibit typical characteristics of the type A PLC effect. The geometric compatibility factor m′ is used to describe the deformation compatibility between two adjacent grains, and is expressed as [32,33]:

(2) $$m^{\prime }=\;\cos\psi \cdot \cos k$$

where ψ is the angle between the normal vectors of two adjacent grain slip planes, and κ is the angle between the shear directions of two adjacent grain slip planes. The value of m′ typically ranges from 0 to 1, and the closer it is to 1, the easier it is for a slip to occur between two adjacent grains, which facilitates the continuous transmission of dislocations across grain boundaries, thereby effectively alleviating local stress concentration and enhancing the overall ductility of the joint [32]. In this study, m′ > 0.7 is designated as an indicator of excellent deformation compatibility between two adjacent grains. The Schmid factor is a quantitative parameter that describes the propensity of a specific slip system in crystalline materials to undergo plastic deformation, and its expression is defined as [34,35]:

(3) SF=cos∅·cosλ

SF represents the Schmid factor, ϕ denotes the angle between the slip direction and the applied load direction, λ is the angle between the slip plane normal and the applied load direction. The Schmid factor is used to predict the ease of activation of a slip system under given loading conditions; a higher Schmid factor indicates that the slip system is more readily activated.

**Figure 14:**
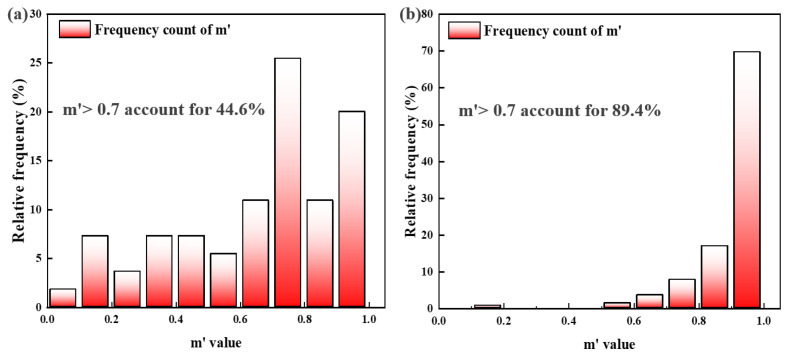
Distribution of the geometric compatibility factor: (**a**) W-temper welded sheet; (**b**) PH welded sheet.

**Figure 15:**
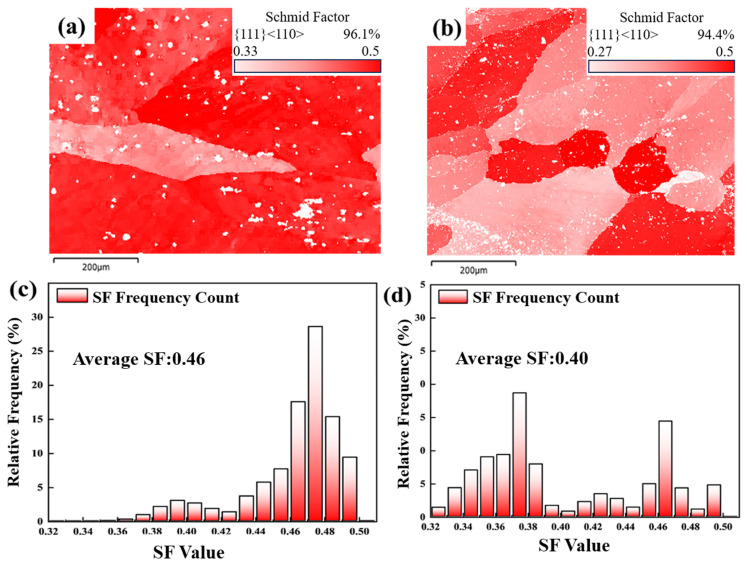
Schmid factor distribution diagrams: (**a**,**c**) W-temper welded joint; (**b**,**d**) PH welded joint.

Figure 14 shows the distribution of m′ values for the tensile fracture specimens of the W-temper welded joints and PH welded joints, while Figure 15 presents a schematic and the distribution of the Schmid factor for the tensile fracture specimens of both joint types. Statistical results indicate that the average Schmid factor for the W-temper welded joints was 0.46, whereas it was 0.40 for the PH welded joints. Based on the Schmid factor distribution, the slip systems in the W-temper condition should be more readily activated than those in the PH welded sheets. This is consistent with the findings of Ji Liu et al. [36]. According to the distribution of the geometric compatibility factor m′, 89.4% of PH welded joints have m′ values exceeding 0.7, while only 44.6% of W-temper welded joints reach this threshold. This indicates that the grain deformation compatibility in PH welded joints is higher than in the W-temper joints.

Research by Xin et al. [37] indicates that a combination of high SF and high m′ promotes the formation of twin pairs in magnesium alloys, thereby improving their elongation. Therefore, the influence of microstructure on ductility must consider both the Schmid factor and the geometric compatibility factor. For W-temper welded joints, the Schmid factor measured after tensile deformation was very high, indicating that the tensile process stored considerable energy for grain boundary sliding. However, only 44.6% of the W-temper joints exhibited a geometric compatibility factor m′ greater than 0.7, suggesting that few regions underwent grain boundary sliding by the end of deformation. Nonetheless, due to the energy accumulated during deformation, most regions that did not experience grain boundary sliding still displayed high Schmid factors. For PH welded joints, the proportion of regions with m′ greater than t was even higher, which indicates that extensive grain boundary sliding occurred during tensile deformation. Due to the higher geometric compatibility factor, multiple slip systems may have been activated simultaneously, and the activity of these different slip systems induced dislocation movement. As these dislocations moved and interacted within the crystal, they formed entanglements. The dislocation entanglements can serve as the cores of sub-grain boundaries. With further deformation, sub-grains develop at grain boundaries and consume dislocations, thereby improving ductility. It should be noted that since most of the slip had already occurred, the Schmid factor in these specimens was relatively low.

## 5. Conclusions

This research evaluated how friction stir welding (FSW) parameters dictate the mechanical integrity and formability of PH 2219 aluminum alloy. The key findings are summarized below.

Optimal parameter selection: The mechanical performance and formability of PH welded sheets are primarily controlled by the welding forward speed. To achieve a peak ultimate tensile strength (UTS) of 459 MPa and an Erichsen formability value of 10.9 mm, a lower forward speed of 50 mm/min paired with a moderate rotational speed of 1000 rpm is recommended.

Microstructural mechanisms: The synergy between high strength and ductility stems from the presence of fine, dispersed θ″ and θ′ precipitates within the weld zone. These precipitates effectively obstruct dislocation movement without inducing detrimental stress concentrations. Furthermore, superior geometric compatibility triggers multiple slip systems, leading to sub-grain formation that accommodates dislocations and boosts overall ductility.

Industrial application and scaling: The findings of this study provide a technical foundation for the aerospace and automotive industries. The PHF process demonstrates significant advantages for industrial applications; it successfully overcomes the bottleneck of the “strength–ductility trade-off” in materials, offering excellent formability while maintaining high strength, thereby effectively meeting the demands of secondary forming for complex components. Future research should incorporate direct thermal measurements and explore a wider range of thicknesses to further validate the applicability and stability of PHF processes at an industrial scale.

## Figures and Tables

**Table 1:** Chemical composition (wt.%) of 2219 aluminum alloy.

Element	Al	Si	Fe	Cu	Mn	Zn	Ti	V	Zr
Content (wt.%)	bal	0.38	0.12	5.5	0.26	0.15	0.04	0.064	0.08

**Table 2:** FSW test parameters.

Serial Number	Rotational Speed w (rpm)	Forward Speed v (mm/min)	Specimens Number
#1	500	50	500–50
#2	500	100	500–100
#3	500	150	500–150
#4	1000	50	1000–50
#5	1000	100	1000–100
#6	1000	150	1000–150
#7	1500	50	1500–50
#8	1500	100	1500–100
#9	1500	150	1500–150

**Table 3:** Heat index values under different welding parameters.

Welding Parameters	500–50	500–100	500–150	1000–50	1000–100	1000–150	1500–50	1500–100	1500–150
HI	5000	2500	1667	20,000	10,000	6667	45,000	22,500	15,000
Level	Lower	Lower	Lowest	High	Medium	Medium	Higher	Higher	Medium

## Data Availability

The original contributions presented in the study are included in the article. Further inquiries can be directed to the corresponding authors.
